# Plant-derived antivirals against hepatitis c virus infection

**DOI:** 10.1186/s12985-018-0945-3

**Published:** 2018-02-13

**Authors:** Ana Carolina Gomes Jardim, Jacqueline Farinha Shimizu, Paula Rahal, Mark Harris

**Affiliations:** 1 Laboratory of Virology, Institute of Biomedical Science, ICBIM, Federal University of Uberlândia, Avenida Amazonas, Bloco 4C – sala 216. Umuarama, Uberlândia, MG CEP: 38405-302 Brazil; 2 Genomics Study Laboratory, São Paulo State University, São José do Rio Preto, SP Brazil; 3 School of Molecular and Cellular Biology, Faculty of Biological Sciences and Astbury Centre for Structural Molecular Biology, University of Leeds, Leeds, LS2 9JT UK

**Keywords:** Hepatitis C, Natural compounds, Antivirals

## Abstract

Hepatitis C virus (HCV) infection is a worldwide public health burden and it is estimated that 185 million people are or have previously been infected worldwide. There is no effective vaccine for prevention of HCV infection; however, a number of drugs are available for the treatment of infection. The availability of direct-acting antivirals (DAAs) has dramatically improved therapeutic options for HCV genotype 1. However, the high costs and potential for development of resistance presented by existing treatment demonstrate the need for the development of more efficient new antivirals, or combination of therapies that target different stages of the viral lifecycle. Over the past decades, there has been substantial study of compounds extracted from plants that have activity against a range of microorganisms that cause human diseases. An extensive variety of natural compounds has demonstrated antiviral action worldwide, including anti-HCV activity. In this context, plant-derived compounds can provide an alternative approach to new antivirals. In this review, we aim to summarize the most promising plant-derived compounds described to have antiviral activity against HCV.

## Background

Hepatitis C virus (HCV) infection is a worldwide public health burden and it is estimated that 185 million people are or have previously been infected worldwide, representing almost 3% of the global population. Most infections persist and chronically infected individuals have a high risk to develop liver cirrhosis and hepatocellular carcinoma after 10 to 30 years of infection [1–3].

HCV transmission occurs by parenteral via. The main route of transmission was blood transfusion until in the 1990s when Food and Drug Administration (FDA) authorized medical centers to screen blood for HCV. Nowadays, the sharing of contaminated syringes by drug users and inadequate sterilization of medical equipment are the main transmission routes [4–6].

There is no effective vaccine for prevention of HCV infection, however, a number of drugs are available for the treatment of infection. Until recently, the standard therapy was based on pegylated interferon (IFN) plus ribavirin (RBV), resulting in a sustained virological response in approximately 50% of patients infected with HCV genotypes 1a/1b and 80% of those infected with genotypes 2 or 3 [7–9]. The availability of new, direct-acting antivirals (DAAs) targeting the NS3/4A protease, NS5B polymerase and NS5A protein have dramatically improved therapeutic options for HCV genotype 1 [10, 11]. However, the high costs and potential for development of resistance presented by existing treatment demonstrate the need for the development of more efficient new antivirals, or combination of therapies that target different stages of the viral lifecycle. The efforts to develop innovative anti-HCV drugs are challenged by the viral high mutation rate, which allows the rapid emergence of resistant strains to DAAs directed to the NS5A and NS3 regions. Further issues to overcome include the development of a drug which can impair the virus with limited side effects on the host cell and the affordable access to care in developing countries.

Over the past decades, there has been substantial study of compounds extracted from plants that have activity against a range of microorganisms that cause human diseases. Natural compounds have received increasing attention for their therapeutic potential, including antiviral properties [12, 13]. An extensive variety of natural compounds has demonstrated either antiviral action worldwide, including anti-HCV activity [2, 12–14], or hepatoprotective effect as described for naringenin, (−)-epigallocatechin-3-gallate (EGCG), silymarin and caffeine [15–18].

In this context, plant-derived compounds can provide an alternative approach to new antivirals. Natural compounds present characteristics such as high chemical diversity, lower cost of production and milder or inexistent side effects than conventional treatment [19]. Additionally, most of the drugs used today in the clinic were first discovered from plants and microorganisms [20–22]. In this review, we aim to summarize the most promising plant-derived compounds described to have antiviral activity against HCV.

### The HCV replication cycle

HCV is a small, enveloped virus, grouped within the genus *Hepacivirus* of the Flaviviridae family [23]. Its single-strand positive genomic RNA of 9600 kb in length has a single open reading frame, with untranslated regions (UTR) in both 5′ and 3′ ends. These UTR regions are well conserved RNA structures essential for the translation of viral proteins and viral genome replication [24, 25].

The HCV viral particles are associated with low density lipoproteins (LDL) and very low-density lipoproteins (VLDL) and this association is related to infectivity. Virions circulate in the blood-stream of patients as lipoviroparticles [26–28]. The first stage of HCV infection is the attachment of virus particles on the surface of the host cell followed by the specific interaction of the viral envelope glycoproteins E1 and E2 with cellular receptor molecules, such as the tetraspanin CD81, scavenger receptor class B type 1 (SRB1), and the tight junction components claudin 1 (CLDN1) and occludin (OCLN) [29]. HCV entry into the host cells by clathrin-mediated endocytosis [30]. The viral RNA is released into the cytoplasm after the uncoating of the nucleocapsid and is translated at the rough endoplasmic reticulum (ER) under the control of the viral internal ribosome entry site (IRES) giving rise to a single polyprotein which is co- and posttranslationally cleaved into 10 proteins: the structural proteins core and envelope glycoproteins E1 and E2, the viroporin p7 and the non-sctructural proteins NS2, NS3, NS4A, NS4B, NS5A and NS5B [31, 32]. To process HCV structural proteins a minimum of two cellular peptidases are required, while the non-structural proteins are cleaved by viral peptidases [33]. The non-structural viral proteins NS3/4A, NS4B, NS5A and NS5B assemble and interact with host proteins to form the virus replication complexes that will amplify viral RNA via the prior synthesis of negative strand intermediates. HCV replication occurs in the host cell cytoplasm in a specialized membranous compartment from the endoplasmic reticulum, termed the membranous web. To form this compartment, viral proteins induce a rearrangement of the ER membranes [34–36]. Host proteins as cyclophilin A, VLDL/LDL pathways and mRNA-122 seem to be associated with the improvement of HCV replication cycle [28, 37]. The assembly of new viral particles occurs in close vicinity to lipid droplets and the core protein interacts with NS5A to recruit nascent genomes. Apolipoprotein E also seems to be essential to the formation of lipoviroparticles [27, 38–41] and modifications observed in E1 and E2 proteins of virions indicate that these proteins are processed by Golgi complex [42].

## Plant-derived compounds with anti-hcv properties

### Blockade of HCV entry

Viral entry is an attractive point of intervention to prevent virion access into non-infected cells. This multistep process can be inhibited by small molecules, including compounds extracted from natural sources that have shown to abrogate HCV infectivity. Unlike for example HIV, no entry inhibitor has been licensed for the treatment of HCV infected patients. Efficient inhibition of this stage of virus life cycle can play an important role in a future combined therapy which could include compounds with different targets and modes of action.

The blockade of HCV entry by the proteins griffithsin (GRFT) and scytovirin (SVN) originally isolated from red [43] and blue-green algae [44], respectively, was reported by Takebe et al. [45]. By their unique structural characteristics, these proteins are able to bind to multiple carbohydrate moieties [46–48] and demonstrated to inhibit HCV in both cell culture and pseudoparticle assays by targeting HCV envelope glycoproteins E1 and E2. The authors first observed an effect of these proteins using a replicon assay system and a potent activity against HCV in a cell-based assay with JFH-1 virus (SVN EC50 17 nM, GRFT EC50 0.4 nM; CC50 = 34 mM and a selectivity index (SI) of 84,000). The results were expanded upon utilizing chimeras encompassing the E1 and E2 proteins from the HCV 2a isolate J6 or the HCV 1b isolate TH, showing that multiple viral isolates were sensitive to both GRFT and SVN. Further, by using HCV pseudoparticle assay bearing E1 and E2 proteins from the HCV 2a isolate J6 and the HCV 1b isolate TH, they demonstrated that GRFT (EC50’s ranging from 0.3–14.1 nM) and SVN (EC50’s ranging from 3.2–96 nM) acted early in the viral lifecycle, inhibiting all three HCV isolates. They were able to demonstrate that these proteins activity on HCV entry was explained by their targeting of high-mannose oligosaccharides on either E1 or E2, but did not inhibit binding of E2 to CD81. In addition, they showed that GRFT was active in an albumin-urokinase plasminogen activator/severe combined immunodeficient chimeric mouse model of HCV infection with up to a 2.5-log reduction of viral titers. The authors also tested GRFT in vivo by using a transgenic mouse model. The dose of 20 mg/kg/day of GRFT was administered for a total of 18 days and at the end of the experiment a reduction of _~_ 2.7 log in HCV levels was observed in serum.

Recently, a study which evaluated the anti-HCV properties of *Limonium sinense* extracts demonstrated that an aqueous extract from the underground part of the plant (LS-UW) potently inhibited HCV early entry events by impairing virus attachment and entry/fusion without affecting viral replication, translation and cell-to-cell propagation [49]. They evaluated the bioactive molecules in LS-UW that could contribute to its anti-HCV effect and identified gallic acid (GA) as the compound which most inhibited HCVcc infection (EC50 = 24.31 ± 6.90 μM; CC50 = 346.59 ± 27.44 μM; SI = 14.25). GA also targeted early viral entry, specifically inactivating cell-free virions and blocking virus binding to the host cell surface, with little effect against the post-binding viral entry/fusion, showing that further compounds may be acting on this stage. Additionally, interaction(s) between constituents could result in synergistic effects to exert anti-HCV efficacy observed by treatment with LS-UW. The authors demonstrated that both LS-UW and GA could also suppress HCV infection of primary human hepatocytes. However, the specific mechanisms by which LS-UW and GA act was not clarified.

The inhibition of early steps of HCV life cycle was also observed by the treatment of HuH 7.5 cells infected with Gaussia luciferase reporter viruses (Jc1-GLuc) with a saponin-rich methanolic extract of *Bupleurum kaoi* roots (BK) or its associated saikosaponins SSa, SSb2, SSc, and SSd [50]. *Bupleurum* spp. roots (Radix Bupleuri) are frequently used as herbal treatments for liver diseases and saikosaponins showed to attenuate liver fibrosis [51]. By adding compounds at different times to the infectious HCV culture system Lin et al. demonstrated that SSb2 at 50 μM was able to block infection by neutralizing free virus particles and abolishing viral attachment and entry/fusion. In contrast, BK (50 μM) and SSa (8 μM) appeared to be most effective during the entry/fusion event [50]. The molecular mechanism of action of SSb2 is related to the antiviral effect of this compound on HCV glycoproteins during the initial stages of infection. The pan-genotypic antiviral activity was also demonstrated using chimeric HCV particles from genotypes 2b (J8/JFH1), 3a (S52/JFH1), and 7a (QC69/JFH1), and clinical HCV isolates of different genotypes. These data provide evidence that SSb2 may act as an efficient inhibitor of HCV viral entry in vitro*.*

Haid et al. screened a library of natural phenolic compounds isolated from extracts of *Marrubium peregrinum* *L (Lamiaceae)* for their anti-HCV activity [52]. By using the highly permissive human hepatoma cell line Huh7-Lunet/CD81 with firefly luciferase reporter viruses based on the intragenotypic genotype 2a chimera Jc1, they identified an active extract. Ladanein was the dominant flavonoid and demonstrated to possess the antiviral activity by treatment of cells with natural or synthesized compound (BJ486K) that was based on the natural chemical structure. Natural ladanein and BJ486K inhibited a post-attachment entry step, but not RNA replication or assembly. BJ486K, similar to CD81-specific antibody used as one of the HCV entry control inhibitors, was active not only during HCV inoculation but also after virus binding, it was shown to be effective against all major HCV genotypes (1a, 1b, 2b, 3a, 4a, 5a, and 6a) with an EC_50_ ≤4.2 μM, including a variant (mutation G451R within the HCV envelope protein E2) [53] that is resistant to the entry inhibitor ITX5061 by reducing viral dependency on SR-BI-receptor [54], indicating that it inhibits HCV cell entry independently of viral genotype/subtype. The antiviral activity of BJ486k was also demonstrated in primary human hepatocytes in a dose dependent manner with an EC_50_ of approximately 10 μmol/L. Additionally, combined administration of BJ486K and cyclosporine A showed a synergistic effect in inhibition of HCV infection. The oral bioavailability of BJ486K was stablished in mice, with plasma level of 329 nmol/L after a single oral dose of 0.25 mg/kg. However, the effects of BJ486K against HCV in vivo have not been tested [52].

Delphinidin, a polyphenol plant pigment that gives a blue-purple color to flowers and berries, was reported as an entry inhibitor against all HCV genotypes [55]. Delphinidin exhibited strong inhibition of infection (EC_50_ of 3.7 ± 0.8 μM), The effects of delphinidin were evaluated by adding the compound to the cells prior to, during and after infection. However, the antiviral activity was only observed during infection, suggesting an activity of delphinidin on the virus particle. The results of a binding assay demonstrated that delphinidin reduced HCV attachment by decreasing the amount of HCV RNA bound to the cell surface. However, delphinidin had no effect on virus aggregation or disrupted the envelope of virus particle. The authors also investigated whether delphinidin affected the morphology of virus particle by incubating HCVpp (genotype 2a) with delphinidin or controls and cryoTEM analysis. They demonstrated that this polyphenol acts on E1 and E2 glycoproteins inducing conformational changes on the viral particles, affecting interaction between the viral particles and cell surface.

### Interference with HCV replication

Inhibiting HCV replication by blocking the synthesis of newly viral genomes is a promising strategy for the development of new antivirals. Targeting this step of HCV life cycle specifically abrogates the production of viral genomes in HCV infected cells.

The inhibition of HCV replication by plant-derived compounds was previously described. In 2014, Choi et al. reported that xanthone compounds identified within ethanol extract from mangosteen frit peels (MG-EtOH) suppressed HCV replication [56]. *Garcinia mangostana* L., commonly known as Mangosteen, belongs to the Clusiaceae family and is cultivated manly in Indonesia, Malaysia, Philippines and Thailand. MG-EtOH was able to block HCV genome replication by reducing both protein and RNA levels in infectious replicon systems of genotypes 1b (EC_50_ 5.1 μg/mL) and 2a (EC_50_ 3.8 μg/mL). An additive effect of MG-EtOH and the NS5A inhibitor daclatasvir [57] was also confirmed [56]. It was also shown that MG-EtOH had a modest inhibitory effect on NS5B in vitro and was able to restore the increasing levels of reactive oxygen stress (ROS) production by HCV infection to normal levels. α- and β-mangostins (EC_50_ 6.3 μg/mL and 2.7 μg/mL) were known to be the molecules responsible for inhibitory effect on HCV.

The reduction of protein and RNA levels was also shown by the treatment of subgenomic replicon harbouring cells with 3-hydroxy caruilignan C (3-HCL-C) isolated from *Swietenia macrophylla* stems, from the Meliaceae family, used as a folk medicine in Malaysia. 3-HCL-C exhibited high anti-HCV activity at nontoxic concentrations, with an EC50 value of 10.5 ± 1.2 μM. Additionally, combinations of 3-HCL-C and interferon-α (IFN-α), HCV NS5B polymerase inhibitor (2′-C-methylcytidine; NM-107) or an HCV NS3/4A protease inhibitor (Telaprevir; VX-950) increased the suppression of HCV RNA replication. The mechanism by which 3-HCL-C interfered with HCV replication was shown to involve induction of IFN-stimulated response element transcription and IFN-dependent anti-viral gene expression [58].

Plumbagin (5-hydroxy-2-methyl-1,4-naphthoquinone) is mainly found in roots of *Plumbago indica* *L., a* plant of the Plumbaginaceae family. Plumbagin has demonstrated several biological activities including anticancer [59], anti-inflammatory and antimicrobial [60]. Hassan et al. reported a dose-dependent effect of plumbagin on HCV lifecycle by the infection of Huh-7.5 cells with the full-length HCV FL-J6/JFH/JC1 for 6 h and treatment with plumbagin for 96 h. The intracellular HCV RNA was quantified by real-time PCR and plumbagin demonstrated an IC50 value of 0.57 μM/L and SI = 53.7 [61]. The authors analyzed the expression of non-structural protein NS3 of HCV and the cellular protein hA3G, a cytidine deaminase that was described as a host factor with anti-HCV activity [62]. Results demonstrated that plumbagin increased the expression of hA3G protein and decreased NS3 protein expression in a dose-dependent manner.

Xanthohumol (XN) is the main prenylated chalcone present in hops (*Humulus L.*, Cannabaceae) [63, 64]. This natural product has been shown to possess biological properties including anticancer [65], anti-inflammatory [66, 67] and antiviral activities [68–70]. Lou et al. demonstrated that XN at 7.05 or 14.11 μM achieved an inhibitory effect on HCV replication in vitro similar to that of IFN- α 2b by measuring luciferase activity and RNA levels in Huh7.5 cells infected with J6-JFH1 isolate of HCV [71]. The mode of action of XN on HCV infection remains to be elucidated. However, author suggested that XN may act as a host-targeting inhibitor by affecting the triglyceride-synthesizing enzyme diacyglycerol acyltransferase-1 (DGAT1) expression or/and the microsomal triglyceride transfer protein (MTP) activity, based on previous evidence [72–74]. Yang and coworkers administrated 10 mg/ml of XN in *Tupaia belanger*i infected with HCV. The XN was divided into 3 doses and given to the animals by gavage three times a day for 12 weeks. The results demonstrated a decrease in liver damage and modulates oxidative reaction and apoptosis in animals treated with XN [75].

The flavonoid Apigenin, which is present in many fruits and vegetables, also has diverse biological effects, including improvement of the cancer cell response to chemotherapy [76], tumorigenesis [77, 78], modulating immune cell function [79], and anti-platelet activity [80]. Apigenin is also demonstrated to have inhibitory effect on the maturation of a subset of miRNAs and on the subsequent miRNA functions [81]. MicroRNA122 (miR122), abundantly expressed in the liver and essential for the HCV RNA stability and propagation, positively regulates HCV replication through binding to the 5’-UTR of the HCV genome [82, 83]. In 2014, Shibata et al. demonstrated that 5 μM of Apigenin inhibited HCV replication in vitro without affecting cell growth or viability. The inhibitory effect of apigenin on HCV replication was possibly explained by decreasing the levels of mature miR122 through the inhibition of the phosphorylation of TRBP, a component of miRNA-generating complexes via impaired mitogen-activated protein kinase activation [84].

Coffee intake has been associated with lower rates of liver disease progression in chronically HCV infected patients [85]. Several compounds extracted from coffee, including caffeine, may have beneficial effects on the liver [86–88]. Caffeine has been demonstrated to have an antiproliferative effect on liver cancer cells [89], may limit liver fibrosis [90] and interact with cellular pathways to slow the progression of liver diseases [91]. The effects of caffeine on HCV replication was evaluated in 2015 by Batista et al. by using a cell line stably harbouring the SGR-Feo JFH-1 and by infecting Huh-7.5 cells with J6/JFH1 HCV isolate [92]. Caffeine efficiently inhibited HCV replication in a dose dependent manner and reducing HCV RNA levels and protein expression by up to 80% of the non-treated control at the highest safely tolerated concentration (2 mM). The half-maximal inhibitory concentration (IC_50_) was reported to be in order of 0.726 mM. The authors demonstrated that cell cycle progression pattern and apoptosis levels remained unchanged in caffeine treated cells. Induction of autophagy was also investigated but no significant difference was found apart from a higher proportion of cells in an autophagic state observed in the presence of caffeine.

Compounds extracted from Brazilian plants also showed to dramatically inhibit HCV replication in vitro. The compounds APS (EC_50_ = 2.3 μM), a natural alkaloid isolated from *Maytrenus ilicifolia*, the tetrahydrofuran lignans 3*43 (EC_50_ = 4.0 μM) and 3*20 (EC_50_ = 8.2 μM) and the secolignan 5*362 (EC_50_ = 38.9 μM) from *Peperomia blanda* were tested by their inhibitory effects on HCV replication using either subgenomic reporter SGR-Feo-JFH1 and the full-length Rluc-J6/JFH1. Data demonstrated that by treatment with these compounds dramatically reduced HCV RNA and protein levels and acted mainly by preventing the formation of new replication complexes [93]. It was also shown that replication blockade by Brazilian naturally occurring compounds was independent of HCV genotype and was not affected by variants described to confer resistance to the NS5A targeting compound daclatasvir [57, 94, 95]. At the same work, a natural kavalactone F8*40 isolated from *Piper fuligineum*, showed a significant but not dramatic effect on IRES-directed translation, correlated with a reduction of protein levels in the presence of this compound, suggesting that its mode of action is related to the inhibition of IRES-mediated translation [93].

The effect on baseline IRES translation was earlier showed by treating of cells with quercetin, a plant-derived flavonoid present in fruits, vegetables, leaves and grains [96]. The inhibition of HCV replication by quercetin was first demonstrated by Gonzalez et al. in 2009 [97]. These researchers sought to identify interaction between HCV NS5A and cellular protein that could play a role in the virus production. By co-immunoprecipitation and co-localization assays they detected interactions between NS5A and the heat shock proteins (HSP) HSP40 and HSP70. Using a transient transfection luciferase-based tissue culture system, the authors demonstrated that treatment with quercetin, an inhibitor of HSP synthesis, markedly reduced HCV IRES activity and its increase by NS5A. However, quercetin was found to decrease IRES activity either in absence or in presence of NS5A. Quercetin treatment (50 μM) did not reduce viral genome copy numbers but NS5A protein levels and had strongly inhibited HCV production in cell culture. RNA replication of SGR -harboring cells was not affected by treatment, supporting that the major role of HSP40 and HSP70 may not be in genome replication but rather in subsequent stages in the viral life cycle. Additionally, the effects of quercetin could be related to a global reduction in most of the HSP family members, not restricted to the HSP40 and HSP70, as siRNA-mediated depletion of HSP proteins had no effect on HCV particle production.

Later in 2012, the same research group further investigated the mode of action of quercetin and other structurally related bioflavonoids [98]. Using a HCV IRES bicistronic reporter assay system, they showed that the translation inhibitory effect of quercetin treatment was NS5A dependent, consistent with the mechanism of NS5A/HSP7 complex-driven IRES-mediated translation of viral proteins. They also demonstrated that quercetin had a lesser effect on intracellular infectious virus assembly and had no effect on virion secretion. Furthermore, quercetin had a strong inhibitory effect on HSP70 expression, supporting the conclusion that bioflavonoids mediate their antiviral effects at least in part by blocking HSP expression and underscore the role of HSPs in HCV life cycle.

Also in 2012, Bachmetov et al. identified quercetin as the active substance responsible for the inhibition of NS3 protease activity by *Embelia ribes* plant extracts [99]. They showed that it was found to inhibit NS3 activity in a specific dose-dependent manner in an in vitro catalysis assay. Using a model system in which NS3 engineered substrates were introduced into NS3-expressing cells, they demonstrated that quercetin-mediated inhibition could be explained by an NS3 protease specific mechanism. This inhibition of NS3 was confirmed in cell culture with NS3 protease-dependent fluorescent reporter systems. They also demonstrated that quercetin at 10 μg/mL inhibited HCV RNA replication up to 70% as analysed in the SGR system, as well as the HCV infectious virus production by treating cells infected with intergenotype HJ3–5 chimeric virus up to 95%. Taking all data available, these authors suggested that the high inhibition efficiency of virus production could be attributed to a mutual inhibitory effect of quercetin targeting both NS3 protease and NS5A.

In addition to these described inhibitory mechanism, Pisonero-Vaquero et al. investigated in 2014 the contribution of oxidative/nitrosative stress (ROS/SNS) and lipid metabolism modulation in the inhibition of HCV replication by quercetin [100]. They showed that quercetin inhibited HCV replication in Huh7 cells expressing full-length genotype 1b HCV replicon in a dose-dependent manner alone (up to 61%, 5 μM), or combined with IFNα (up to 72%, 5 μM), and HCV NS5A and core protein expression was also reduced (up to 78%, 5 μM). Quercetin acted by decreasing HCV-induced ROS/RNS generation (up to 35%, 5 μM) and lipoperoxidation (up to 30%, 5 μM) in replicating cells. The treatment significantly decreased HCV-induced intracellular lipid accumulation in a dose-dependent manner (Q5: 55% vs HCV-G1 vehicle-treated cells), inhibited liver X receptor (LXR)a-induced lipid accumulation in LXRa-overexpressing and replicon-containing Huh7 cells (Q5: 34% vs HCV-G1 vehicle-treated cells). Inactivation of phosphatidylinositol 3-kinase (PI3K)/AKT pathway was inferred to be the mechanism involved in modulating LXRa-dependent lipogenesis by quercetin and may contribute to the inhibition of viral replication.

An additional anti-HCV activity by quercetin-based treatment was recently suggested [101]. Lulu et al. predicted the 3D structure HCV NS2 protein and performed docking studies to identify phytochemical compounds which could have strong affinity towards its protease. Ten plants-derived compounds were tested and quercetin revealed minimum binding energy of 7.95 kcal/mol with NS2, demonstrating the theoretical potential of this compounds to target HCV NS2 protease.

The inhibitor activity of NS3/4A protease was also shown by testing triterpenes from *Cynomorium songaricium* [102]. “SuoYang” (*Cynomorium songaricum* Rupr; Cynomoriaceae) is a parasitic plant, which grows mainly in the northern part of China, such as Inner Mongolia Autonomous Region and Gan Su Province. Using an HCV protease assay it was shown that malonyl ursolic acid hemiester tested triterpen was the most potent inhibitor of NS3/4A in this free-cells method.

### Assembly and release

Naringenin is a dietary supplement derived from naringin, one of the most abundant flavonoids in citrus fruits. It has been demonstrated to possess anti-oxidant, anti-inflammatory, anti-carcinogenic properties, and also to reduce cholesterol levels both in vitro and in vivo [103–107]. Grapefruit’s bitter taste is caused by the presence of naringin, which is metabolized into naringenin. It was previously demonstrated that Naringenin, or its glycosylated form, can reduce cholesterol levels in animals and in human cells both in vivo and in vitro [108, 109].

In 2008, Nahmias et al. demonstrated a dose-dependent inhibitory effect of naringenin on HCV release [74]. The authors treated infected Huh-7.5.1 cell with naringenin for 24 h, supernatant was collected, cells were harvested and HCV RNA was measured by qRT-PCR. The results showed a decrease of HCV core secretion and extracellular HCV positive strand RNA, demonstrating that naringenin at the concentration of 200 μM could inhibit up to 80% HCV secretion. Later, Goldwasser et al. related that naringenin was able to block the assembly of intracellular HCV particles [110]. The authors suggested that the mode of action of naringenin could be the PPARα activation. PPARα is a transcription factor associated with lipid metabolism in the liver, related to the reduction of lipogenesis and VLDL secretion [111], and these mechanisms are extremely related to HCV infectivity due to affect the viral assembly. By treating Huh7.5.1 cells infected with JFH1 virus with 200 μM naringenin or 10 μM of a classical PPARα agonist (WY14643), they were able to demonstrate that naringenin or WY14643 caused an inhibition of HCV RNA secretion and did not affect the intracellular levels of HCV RNA. Naringenin was also co-incubated with WY14643 and no increased effect was observed, demonstrating the possible effect of naringenin in PPARα activation. More recently, Lulu et al. analysed by molecular docking studies the structural interactions between naringenin and the protein NS2 protease [101]. They demonstrated that naringenin exhibited a lower minimum binding energy (− 7.97 kcal/mol) than RBV (− 5.89 kcal/mol) to bind to NS2 protein, suggesting that naringenin requests a lesser amount of energy to disrupt HCV NS2 protein. These results demonstrated a theoretical potential of naringenin as an inhibitor of HCV NS2 protease [112]. However, this data has not been confirmed by in vitro or in vivo assays yet.

### Broad spectrum activity against HCV

Honokiol, a lignan isolated from leaves of *Magnolia officiais*, was shown to have multiple effects on HCV infection, strongly inhibiting HCV infection (EC_50_ 1.2 μM) in both the J6/JFH1 reporter virus system (p7-Rluc2A) and in a pseudo-particles system containing glycoprotein of three different HCV genotypes (1a, 1b and 2a) [100–102]. The mechanism in which honokiol affects HCV entry was reported by Lan et al. [113]. The treatment of cells with this natural compound suppressed the OCLN and SR-BI receptors involved in HCV infection. The authors also demonstrated that honokiol was able to inhibit HCV IRES-mediated translation. However, this antiviral action was observed only at the highest concentration treatment (30 μM) and no effect was detected at concentration lower than 20 μM. Honokiol was also effective against HCV replication as observed against two SGR, the genotype 1b (pFK-I389luc-NS3–3′/5.1) and genotype 2a (pSGR-Fluc-JFH1).

Oxymatrine and matrine are the two major alkaloids in the aqueous extract from the *Sophora* root. Oxymatrine is reported to have antiviral activity against HCV in cell cultures and has shown hepatoprotective activity in an animal study [114–116]. In a clinical perspective, the components oxymatrine and matrine found in *Sophora* roots have shown to reduce viral load and liver fibrosis [19, 117]. Mao et al. evaluated the efficacy and safety of oxymatrine for the treatment of hepatic fibrosis in patients with chronic viral hepatitis B or C. Patients took three doses of 300 mg oxymatrine a day for 52 weeks. The data obtained demonstrated a decrease of alanine aminotransferase (ALT) and histological inflammatory activity decreased from 46.08 (± 3.84) to 4.00 (± 2.97) after treatment with oximatrine based on Semi-quantitative scoring system [118].

Epigallocatechin-3-gallate is the major component in green tea. This polyphenol belongs to the Flavonoid family and is reported with antiviral activity against a range of viruses such as Chikungunya virus [119], Enterovirus-71 [120] and HCV [55, 121] in addition to other activities as anti-inflammatory and antioxidative [122] anti-cancer [123]. EGCG was initially described by Ciesek et al. as an inhibitor of HCV entry and cell to cell spread. The inhibition of HCV entry was confirmed by incubating EGCG with infected Huh-7.5 in a dose-dependent manner. The results demonstrated the efficient blockage of virus entry with an approximate IC_50_ of 2.5 μg/mL. To demonstrate whether EGCG can inhibit cell to cell spread the authors infected Huh-7.5 with HCVcc in the presence of EGCG and then co-cultured the infected cells with näive Huh-7.5 cells in an agarose overlay assay [124]. EGCG was added to the co-culture and no spread was observed during the treatment with EGCG at 3 μg/mL [125]. Calland et al. described EGCG acting on the early steps of virus entry by disrupting lipid metabolism in the host cells [121]. Recently, the same authors described that EGCG blocks virus infection (EC_50_ of 10.6 ± 2.9 μM) due to its action on E1 and E2 glycoproteins and induces conformational changes in the viral particles, a mechanism which affects the interaction between the viral particles and cell surface, as observed for delphinidin [55].

Chen et al. performed in vitro assays using the cell culture derivative JFH-1 HCV (HCVcc) system with GFP [126] and demonstrated that EGCG strongly decreased HCV infection (EC_50_ of 17.9 μM) in a mechanism related to the virus entry inhibition. However, this group also observed a moderate action of EGCG on HCV replication. The authors used an HCV construct with an in-frame deletion in the HCV E1E2-coding sequence [127], to avoid reinfection. Huh-7.5.1 cells were treated with 80 μM EGCG 4 h post transfection and the HCV RNA levels were measured 72 h later. The results showed that EGCG blocked HCV replication by the reduction of both the HCV RNA levels (66%) and the expression of viral proteins NS3 and NS5A. The same inhibitory effect was also observed for HCV genotype 1b (Con-1 replicon) [128] (reduction of 56,6% of HCV RNA and a decrease of NS3 and NS5) [123]. Taken together this studies demonstrated the extraordinary potential of EGCG against HCV.

Silymarin is a mixture of silybin A, silybin B, isosilybin A, isosilybin B, silychristin, isosilychristin, and silydianin extracted from *Silybum marianum* (milk thistle). This extract has shown recently to block virus entry, RNA and protein expression, virus production and cell to cell spread of virus [129]. Additionally, this compound demonstrated a hepatoprotective effect on treated cells [130]. One of the major components of silymarin is silibinin, a mixture of semi-purified silybin A and silybin B and commercially available.

In 2007, Polyak et al., showed that silymarin was capable of blocking HCV infection using JFH1 [131] and also H77/JFH and J6/JFH systems [129]. The authors demonstrated that silymarin effect could be due to the inhibition of NS5B polymerase activity, although it did not seem to be the main mechanism of action of this compound.

Blaising et al. tested the purified compounds from silymarin extract silybin A, isosilybin A, silybin B, isosilybin B, silibinin, silychristin, taxifolin, isosilychristin and silydianin. The authors evaluated the antiviral activity of these compounds on HCV infection and demonstrated that silibinin is the main inhibitor of HCV fusion in silymarin extract. Using live-cell imaging studies in 3D–confocal microscopy, the authors observed the HCV intracellular trafficking into the host cells and demonstrated that silibinin blocked endosomal traffic in Huh-7.5. HCV entry in host cells is a complex processes which involves the presence of receptors CD81, OCLN, CLDN1 and SRB1 [29]. It is also associated with clathrin receptor that is related to clathrin-mediated endocytosis [30, 132]. Silibinin also inhibited HCV entry in primary human hepatocytes by hindering clathrin-mediated endocytosis and consequently viral entry [133].

### Future perspectives

Most of the plant derived compounds cited in this review showed promising results in in vitro analysis (Table 1). However, some of the challenges in this field are the lack o*f* in vivo studies, bioavailability and pharmacokinetics of plant derived compounds against HCV. In addition, some natural compounds were reported as Pan Assay INterference compoundS (PAINS), which may affect the results obtained by bioassays [134]. Compounds which possess these characteristics, such as quercetin and EGCG, need to be analyzed very carefully. Additionally, further analysis to confirm the effect of the compounds in vivo should be performed and also tests for obtaining the bioavailability and pharmacokinetics. According to the literature, the bioavailability of natural compounds is very diverse. The compounds Ladanein, after a single oral dose of 0.25 mg/kg, demonstrated a bioavailability of 329 nmol/L [52]. A pharmacokinetics study for XN demonstrated that the maximum XN concentrations were 45 ± 7 μg/L, 67 ± 11 μg/L, and 133 ± 23 μg/L for 20, 60, and 180 mg oral dose, respectively [135]. Alternatives to improve the bioavailability of some natural compounds have been described with the advancement of nanotechnology [136], by improving the delivery of these compounds to their targets and raising the half-life of the compound within the body. However, there are only few studies on this topic by now.

**Table 1:** Plant-derived antivirals tested in cellular models with activity against HCV life cycle. Structures from PubChem (https://pubchem.ncbi.nlm.nih.gov)

Compound	Structure	Extracted from	Viral Inhibition step	HCV genotypes	EC50	CC50	Reference
Griffithsin	^a^	*Griffithsia sp*	Entry	1b, 2a	0.4 nM	33.6 μM	[45]
Scytovirin	^a^	*Scytonema varium*	Entry	1b, 2a	17 nM	23.8 μM	[45]
Gallic Acid		*Limonium sinense*	Entry	2a	24.31 ± 6.90 μM	346.59 ± 27.43 μM	
Saikosaponin b2		*Bupleurum kao*	Entry	2b, 3a,7a	16.13 ± 2.41 μM	740.4 ± 28.35 μM	
Ladanein		*Marrubium peregrinum L*	Entry	1a, 1b, 2b, 3a, 4a, 5a, 6a	≤2.54 μmol/L	98.04 μmol/L	[52]
Delphinidin		Anthocyanidin	Entry	1a, 2a	3.7 ± 0.8 μM	–	[55]
Epigallocatechin-3-gallate		*Camellia sinensis*	Entry/Replication	1a, 2a 2a 1b,2a	10.6 ± 2.9 μM 2.5 μg/mL 17.9 μM	232.6 μM	[121] [125] [123]
Xanthone extract	–	*Garcinia mangostana L.*	Replication	1b 2a	5.1 μg/mL 3.8 μg/mL	12.8 μg/mL	[56]
3-hydroxy caruilignan C		*Swietenia macrophylla*	Replication	1b	10.5 ± 1.2 μM	–	[58]
Plumbagin		*Plumbago indica L.*	Replication	2a	0.57 μM	30.65 ± 1.25 μM/L	[61]
Xanthohumol		*Humulus L.*	Replication	2a	–	–	[71]
Apigenin		Flavone	Replication	2a	–	–	[84]
Caffeine -		Alkaloid	Replication	2a	0.726 mM	–	[92]
APS	^b^	*Maytrenus ilicifolia*	Replication	2a	2.3 μM	135.24 μM	[93]
3*43	^b^				4.0 μM	18.8 μM	
3*20	^b^				8.2 μM	32.8 μM	
5*362	^b^	*Peperomia bland*	Replication	2a	38.9 μM	73.91 μM	
Quercetin		*Embelia ribes*	Replication	1a, 2a 1b	–	–	[99] [100]
Ursolic acid		*Cynomorium songaricium*	Replication	-	16 μg/mL	–	[102]
Acetyl ursolic acid					11 μg/mL	–	
Malonyl ursolic acid hemi- ester					8 μg/mL	–	
Glutaryl ursolic acid hemiester					3 μg/mL	–	
Oxalyl ursolic acid hemiester					5 μg/mL	–	
Succinyl ursolic acid hemiester					10 μg/mL	–	
Ursolic acid methyl ester					94 μg/mL	–	
Naringenin		Flavanone	Release/Assembly	2a	- 109 μM	–	[74] [110]
Honokiol		*Magnolia officiais*	Entry/Replication	1a,1b, 2a	1.2 μM	35 μM	[113]
Silymarin Extract	*–*	*Silybum marianum*	Entry/Replication	2a 1a/2a 1b, 3a	- 5 μM 15 ± 4 μM	- -	[131] [129] [133]
Silibinin		*Silybum marianum*	Entry	1b, 3a	*34 ± 3* μM	–	[133]

^a^Structures from RCSB Protein Data Bank (http://www.rcsb.org)

^b^Structures from [93]

## Conclusion

In recent years, the standards treatments against hepatitis C have shown several improvements. However, many issues remain unsolved, as the prevalence of resistance associated variants to DAAs. Natural products represent an increasing source in development of new drugs and could either improve the DAAs treatment or provide alternative approaches in the HCV treatment. Several plant-derived compounds have shown effectiveness against HCV and the improvement on the HCV assay systems allows a better evaluation of the antiviral activity of compounds act on HCV life cycle. However, many mechanisms of action of active compounds remain unknown. Considering the current worldwide biodiversity there may be many plant-derived products to be discovered by their antiviral actions.

## Abbreviations

3-HCL-C: 3-hydroxy caruilignan C
ALT: Alanine aminotransferase
CLDN1: Claudin 1
DAAs: Direct-acting antivirals
DGAT1: Diacyglycerol acyltransferase-1
EGCG: Epigallocatechin-3-gallate
ER: Endoplasmic reticulum
FDA: Food and Drug Administration
GA: Gallic acid
GRFT: Griffithsin
HCV: Hepatitis C virus
HSP: Heat shock proteins
IFN: Interferon
IRES: Internal ribosome entry site
LDL: Low density lipoproteins
LS-UW: *Limonium sinense* aqueous extract
LXR: Liver X receptor
MG-EtOH: Mangosteen frit peels
miR122: MicroRNA122
MTP: Microsomal triglyceride transfer protein
OCLN: Occludin
PAINS: Pan Assay Interference Compounds
RBV: ribavirin
ROS/SNS: Oxidative/nitrosative stress
SRB1: Scavenger receptor class B type 1
SVN: Scytovirin
UTR: Untranslated regions
VLDL: Very low density lipoproteins
